# Prioritization of Surgical, Obstetric, Trauma, and Anesthesia Care in South and Southeast Asian Countries’ Health Planning and Policy‑making: SOTA Care Policies in South and Southeast Asia

**DOI:** 10.5334/aogh.4532

**Published:** 2024-12-11

**Authors:** Saloni Mitra, Ritika Shetty, Shirish Rao, Sweta Dubey, Siddhesh Zadey

**Affiliations:** 1 Association for Socially Applicable Research (ASAR), Pune, Maharashtra, India; 2 OO Bogomolets National Medical University, Kyiv, Ukraine; 3 Seth GS Medical College and KEM Hospital, Mumbai, Maharashtra, India; 4 Department of Pediatrics, SUNY Downstate Health Sciences University, City of New York, New York, USA; 5 GEMINI Research Center, Duke University School of Medicine, Durham NC USA; 6 Dr D. Y. Patil Dental College and Hospital, Dr D. Y. Patil Vidyapeeth, Pune, Maharashtra, India; 7 Department of Epidemiology, Columbia University Mailman School of Public Health, City of New York, New York, USA

**Keywords:** Global surgery, Southeast Asia, South Asia, health policy

## Abstract

*Background:* Lack of policy prioritization of surgical, obstetric, trauma and anesthesia (SOTA) care in South and Southeast Asian countries could be a potential contributor to limited access to care.

*Objective:* To assess the SOTA care prioritization in National Health Policies, Strategies, and Plans (NHPSPs).

*Methods:* We analyzed NHPSPs from twelve South and Southeast Asian countries. These documents are considered the most important health‑related policy statements. Bangladesh was excluded due to a lack of English translations. We searched sixteen electronic documents for a predefined list of keywords. The list included 52 keywords related to SOTA care and 7 keywords unrelated to SOTA care (used as a control). We used the keyword frequency (mentions per keyword or MPK) as a measure to compare prioritization between SOTA care and non‑SOTA care. We further categorized the SOTA care keywords into five different Lancet Commission on Global Surgery (LCoGS) domains and eight subgroups.

*Findings:* Across twelve NHPSPs, MPK value for SOTA care was 54.09 compared with 202.86 for non‑SOTA, with eight NHPSPs having lower MPK values for SOTA care than non‑SOTA keywords. Four NHPSPs had no mentions related to SOTA care financing and information management. Pediatric surgery and anesthesia were among the neglected subgroups.

*Conclusion:* The analysis of South and Southeast Asian NHPSPs suggests that SOTA care issues are not prioritized in most countries. Pathways to greater policy attention include integrating SOTA care into ongoing health policy‑making and planning efforts and developing and implementing specific national SOTA care plans.

## Introduction

Expanding surgical, obstetric, trauma and anesthesia (SOTA) care in low‑ and middle‑income countries (LMICs) could prevent 1.4 million deaths and 77.2 million disability‑adjusted life years (DALYs) annually [1]. To achieve this, developing and implementing national SOTA care plans is necessary [2]. SOTA care planning relies on alignment with existing national health policies and establishing clear pathways for their integration into the broader health programs. This ensures coherence, avoids duplication, and fosters a holistic approach to addressing surgical needs within the larger health system [3].

The National Health Policies, Strategies, and Plans (NHPSPs) harmonize national health goals, foster collaboration, and define priorities. NHPSPs might also influence budget allocation and serve as crucial indicators of national health priorities. The inclusion of SOTA care in NHPSPs demonstrates a country’s readiness for SOTA care planning. Previous NHPSP document reviews have noted low prioritization of SOTA care in sub‑Saharan African countries, neglect toward pediatric surgery in LMICs despite a strong focus on child health overall, and limited prioritization of SOTA care issues in India [4–6].

With more than a quarter of the world’s population and over 1.6 billion people lacking timely access to safe and affordable SOTA care, research on its policy prioritization is lacking for South and Southeast Asia [7]. The current study fills this gap by asking: (a) What is the relative prioritization of SOTA care compared with other healthcare issues in NHPSPs? and (b) How are different domains within SOTA care planning prioritized in these documents?

## Materials and Methods

### Study context

This study included member nations of the South Asian Association for Regional Cooperation (SAARC) and/or World Health Organization South East Asia Regional Office (WHO SEARO): Afghanistan, Bangladesh, Bhutan, India, Indonesia, Maldives, Myanmar, Nepal, People’s Democratic Republic of Korea (mentioned as North Korea throughout), Pakistan, Sri Lanka, Thailand, and Timor‑Leste. Various frameworks, committees, and global commitments have guided national health policymaking and planning in South and Southeast Asian countries (Table 1). Regardless of the diversity in policymaking landscapes, histories, and broader social contexts, the NHPSPs of these countries articulate a shared commitment to achieving universal health coverage.

**Table 1:** NHPSP documents included in the analysis and brief policy contexts of the South and Southeast Asian countries.

COUNTRY	NHPSP DOCUMENT SCREENED	POLICY CONTEXT
Afghanistan	National Health Strategy 2016–2020 for Afghanistan [8]	Afghanistan, emerging from conflict, developed its first NHPSP in 2004, focusing on equity, women and child health, poverty reduction, and rural healthcare [8].
Bhutan	Eleventh Five Year Plan 2013–2018 for Bhutan [9]	Bhutan’s healthcare transformation since the 1960s, with primary healthcare coverage exceeding 90%, showcased its progress towards Millennium Development Goals (MDGs) [10].
India	National Health Policy 2017 for India [11]	India witnessed the establishment of high‑level Planning Commissions or similar bodies. India’s series of five‑year plans from 1951 to 2012, and its three iterations of NHPSPs (1983, 2002, and 2017) significantly influenced its health agenda, with a focus on Universal Health Coverage (UHC and alignment with global declarations and development goals [6]. India has guidelines or policy documents on trauma care and a blood policy [12]. We have previously included the Indian documents in a longitudinal country‑specific analysis [6].
Indonesia	Rencana Strategis Kementerian Kesehatan Tahun 2015–2019 (English transaltion) [13].	In Indonesia, the initial NHPSP drafted in 1945 saw limited implementation until decentralization reforms in 1998, leading to a diverse approach and a focus on Universal Health Coverage (UHC) [14].
Maldives	Health Master Plan 2016–2025 for Maldives [15]	Maldives established a broad vision under its 2006–2015 NHPSP. Since then, Maldives has gradually refined the 2016–2025 emphasizing areas such ase building trust in the healthcare system and promoting equitable access to services [15].
Myanmar	National Health Plan 2017–2021 for Myanmar [16]	Myanmar’s policies have evolved from a centralized socialist model to market‑oriented approaches, reflected in subsequent NHPSPs such as the 1996 Myanmar Health Sector Reform Program and the NHPSP 2016–2025 [17].
Nepal	National Health Policy 2019 for Nepal [18]	Nepal witnessed the establishment of high‑level Planning Commissions or similar bodies. Nepal’s 1991 NHPSP aligned with the Alma Ata Declaration (1978) to focus on expanding basic primary healthcare services [18]. Nepal is in the process of drafting a National Surgical, Obstetric, and Anesthesia Plan (NSOAP); however, the plan draft has yet to be made public.
North Korea/Democratic People’s Republic of Korea	Democratic People’s Republic of Korea Medium Term Strategic Plan 2016–2020 for North Korea [19]	North Korea’s healthcare system, established in 1948, initially prioritized self‑reliance under the Juche ideology. However, the devastating famine of the 1990s prompted increased cooperation with international organizations such as the WHO and UNICEF, leading to participation in international health agreements [20].
Pakistan	National Health Vision 2016–2025 for Pakistan [21]	Since 2001 Pakistan’s policymaking emphasized equity and access, with progress in health insurance coverage and maternal health initiatives, despite challenges in financing [22]. Of note, Pakistan has a recently drafted National Surgical Vision, that is yet to be implemented [23].
Sri Lanka	National Strategic Framework for Development of Health Services 2016–2025 [24] National Health Strategic Master Plan (NHSMP) 2016–2025 on Preventive Services (Volume I) [25] Curative services (Volume II) [26] Rehabilitation Services (Volume III) [27] Health Administration and Human Resources for Health (Volume IV) [28]	Sri Lanka, with an extended history of health policy development since the early 20^th^ century, refined its NHPSP in 2015 to address evolving needs [29]. Sri Lanka also has guidelines on trauma care [12].
Thailand	Twelfth National Economic And Social Development Plan 2017–2021 for Thailand [30]	Thailand witnessed the establishment of high‑level Planning Commissions or similar bodies. Thailand’s NHPSP transformation was bolstered by strategic shifts and the landmark 1978 Primary Health Care Act [31].
Timor Leste	National Health Sector Strategic Plan 2011–2030. The WHO website had no document for Timor Leste. Hence, we derived the Timor Leste document from a literature review [32].	Timor Leste developed its NHPSP in 2004 shortly after the country gained independence in 2002. It emphasizes evidence‑based decision‑making and accelerated implementation, followed by ongoing efforts in public health improvement [33].

### Document selection

We accessed the most recent NHPSPs of South and Southeast Asian countries through the WHO Country Planning Cycle Database [34]. Sri Lanka had five separate volumes for their NHPSP while other countries had one document each. We excluded Bangladesh due to the absence of an English document. Our goal was to assess if SOTA care is prioritized at the apex level of national policymaking and planning; hence, we uniformly included NHPSPs and not other local/sub‑national documents or SOTA care plans, in line with the past studies [35].

### Document screening

We electronically scanned the documents for pre‑specified keywords to record the frequency of their occurrence. Two groups included 52 SOTA care‑related and six non‑SOTA (control) keywords. Previous studies on SOTA care prioritization in the African continent, India, and the integration of pediatric surgery provided the keywords [5, 6, 35]. The SOTA keywords were further grouped into eight subgroups: surgery, trauma, blood, pediatric surgery, obstetric surgery, anesthesia, oncology, and blindness. The non‑SOTA keywords are aligned with communicable diseases and other public health concerns in South and Southeast Asia [6, 35].

Among keyword mentions, we excluded those unrelated to the research scope. For instance, words like “transport” and “blood” were included only when they specifically referred to transporting surgical equipment, referral transport for SOTA care patients, or blood transfusion related to SOTA care cases. We also included all relevant alternatives for keywords with common abbreviations and synonyms. For example, “RTA” or “road traffic accident” were the included keywords but during document reviews, we also found keywords like “road traffic,” “road accidents” and “traffic accidents,” all of which were relevant to trauma care were considered under the umbrella of “RTA.” We excluded the mentions in the document index, glossary, and annexure sections and retained the context (complete sentences or paragraphs). SOTA care‑related mentions were further classified into domains derived from the Lancet Commission on Global Surgery (LCoGS): infrastructure, workforce, service delivery, financing, and information management domains [2].

One investigator (SM) performed primary screening and a second investigator (RS) rescreened random samples of 10% of the total mentions and 5% of domain‑classified mentions. A third investigator (SZ) resolved conflicts to achieve a validation of at least 85% match in both analyses.

### Outcome variables

The relative importance of SOTA and non‑SOTA groups was proxied using the number of mentions per keyword (MPK) in each group (Equation 1). This metric facilitated comparisons between the two groups and also across countries. To assess the relative importance of different LCoGS domains, we examined the absolute number of mentions for SOTA care‑related keywords and the percentage share of SOTA keyword mentions (Equation 2).

$$Relative\;prioritization\;of\;a\;group\;=\;\frac{Number\;of\;mentions\;of\;all\;keywords\;in\;the\;group}{Number\;of\;keywords\;in\;the\;group\;}\;*\;100$$ Equation 1

$$Relative\;prioritization\;of\;a\;domain\;=\;\frac{Number\;of\;mentions\;of\;SOTA\;keywords\;for\;the\;domain}{Number\;of\;SOTA\;keywords\;for\;all\;domains}\;*\;100$$ Equation 2

## Results

### Analysis of mentions

**Figure 1:**
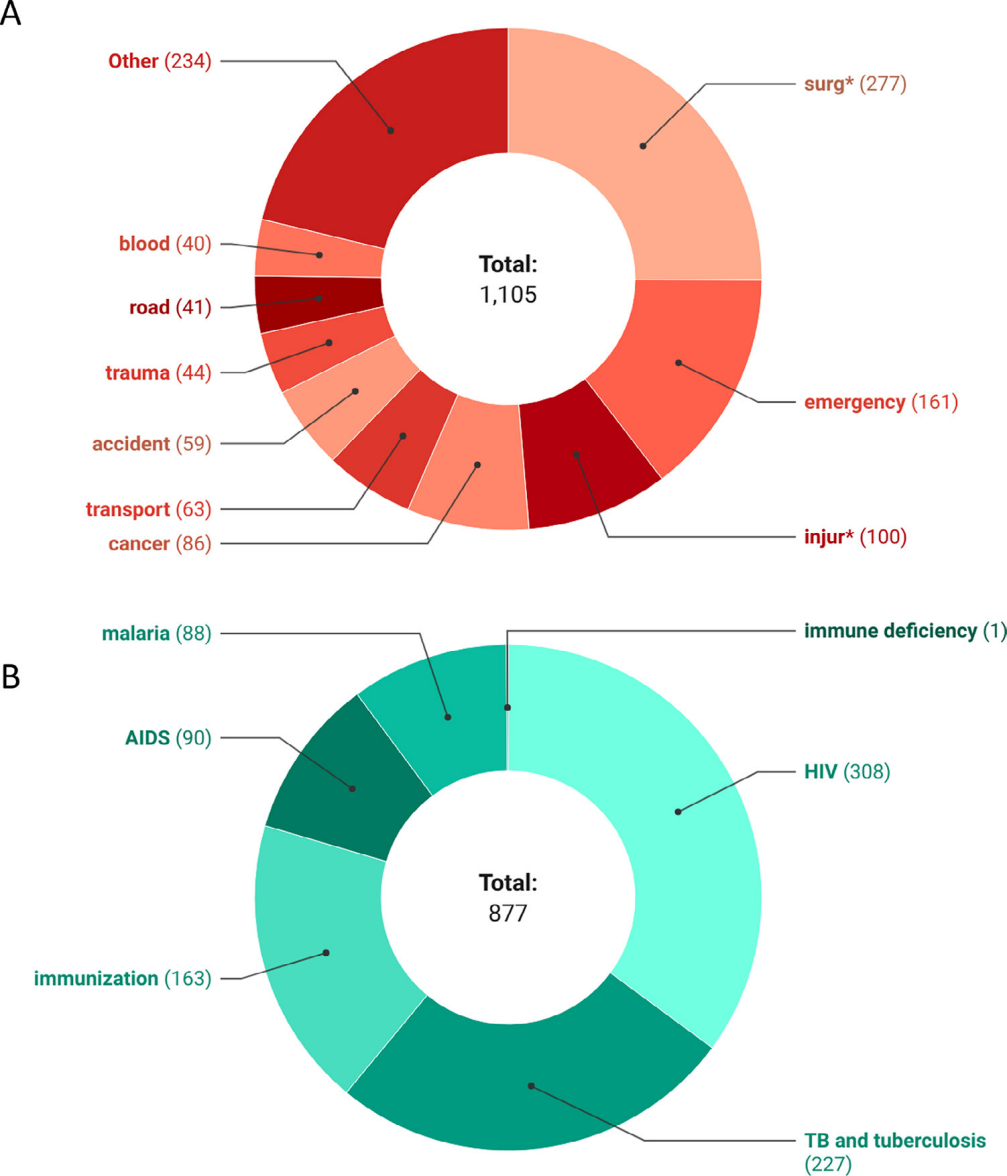
Distribution of SOTA and non‑SOTA mentions across NHPSPs of South and Southeast Asian countries. The ten most frequently mentioned keywords are presented individually, while all other surgical keywords are grouped under “Other.”

Across 16 documents from 12 countries, there were 1105 SOTA and 877 non‑SOTA keyword mentions. Among SOTA keywords, “surg*” had the most mentions (277). In contrast, 19 keywords (operative delivery, open fracture, open fracture fixation, club foot, amputation, incis*, excis*, cauter*, EMONC/emergency maternal, obstetric care with neonatal care, NSV/no‑scalpel vasectomy, *otomy, *stomy, curett*, laparo*, pediatric surg*, hernia, inguinal hernia, circumcision, append*) had no mentions in any of the NHPSPs (Figure 1A and Table 1). On the other hand, all the non‑SOTA keywords were mentioned at least once in NHPSPs, with “HIV” (308) being mentioned the most, followed by TB/Tuberculosis (227) (Figure 1B). Mentions of each keyword in all NHPSP documents are presented in Table 2.

**Table 2:** Keyword‑wise absolute and relative mentions.

KEYWORD	SUBGROUP	NUMBER OF ABSOLUTE MENTIONS	RELATIVE PRIORITIZATION
surg*	Surgery	277	5.33
trauma	Trauma	44	0.85
accident	Trauma	59	1.13
road	Trauma	41	0.79
transport	Other	63	1.21
road traffic accident/road accident/ RTA	Trauma	26	0.5
fall	Trauma	1	0.01
injur*	Trauma	100	1.92
emergency	Trauma	161	3.1
blood	Blood	40	0.77
transfusion	Blood	15	0.29
operat*	Other	19	0.37
OT	Other	10	0.19
OR	Other	1	0.01
operative delivery	Other	0	0
orth*	Other	13	0.25
open fracture	Trauma	0	0
open fracture fixation	Trauma	0	0
club foot	Pediatric surgery	0	0
amputation	Surgery	0	0
wound	Trauma	1	0.02
incis*	Surgery	0	0
excis*	Surgery	0	0
burn	Trauma	2	0.04
cauter*	Other	0	0
obstetric	Obstetric	27	0.52
C‑section, cesarian, caesarean, cesarean	Obstetric	3	0.06
MTP	Obstetric	1	0.02
abort*	Obstetric	21	0.4
EMOC	Obstetric	7	0.13
EMONC	Obstetric	0	0
sterilisation	Obstetric	5	0.1
NSV	Obstetric	0	0
*ectomy	Surgery	5	0.1
*otomy	Surgery	0	0
*stomy	Surgery	0	0
curett*	Obstetric	0	0
laparo*	Surgery	0	0
anaesth*, anesth*	Anesthesia	20	0.38
pediatric surg*	Pediatric Surgery	0	0
hernia	Surgery	0	0
inguinal hernia	Surgery	0	0
circumcision	Surgery	0	0
append*	Other	0	0
cancer	Oncology	86	1.65
neoplasm	Oncology	3	0.06
tumor	Oncology	1	0.02
malignancy	Oncology	1	0.02
chemo*	Oncology	3	0.06
onco*	Oncology	7	0.13
cataract	Blindness	24	0.46
blindness	Blindness	18	0.35
TB and/or tuberculosis	Other	227	37.83
HIV	Other	308	51.33
immune deficiency	Other	1	0.17
AIDS	Other	90	15
malaria	Other	88	14.67
immunization	Other	163	27.17

**Figure 2:**
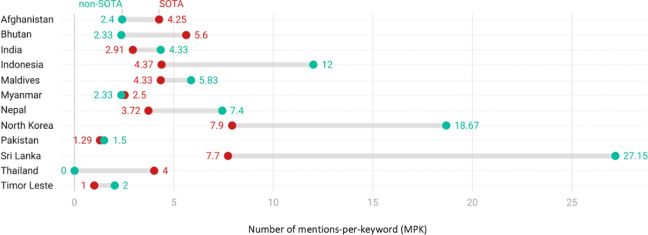
Comparison of SOTA and non‑SOTA MPK values across NHPSPs of South and Southeast Asian countries.

Across all NHPSPs, the SOTA group’s mentions per keyword (MPK) was 54.09 while that for the non‑SOTA group was 202.85—almost over four times greater. NHPSPs of eight countries, namely India, Pakistan, Maldives, Sri Lanka, Nepal, North Korea, Indonesia, and Timor Leste, had lower MPK values for SOTA compared with non‑SOTA keywords (Figure 2). North Korea (7.9) displayed the highest SOTA MPK, followed closely by Sri Lanka (7.7), while Timor Leste (1) had the lowest value. Sri Lanka had the highest non‑SOTA MPK (27.15) while Thailand (0) had the lowest value.

### SOTA care subgroups

The SOTA keywords were categorized under the eight subgroups. Among the eight subgroups of SOTA care keywords, trauma (340) had the most number of mentions followed by surgery (267). Anesthesia (6) had the least number of mentions. Sri Lanka (all volumes combined) had the highest number of mentions of every subgroup, except blood, which had 22 mentions in North Korea’s NHPSP. On the other hand, Timor Leste had the least number of mentions across all subgroups.

### Analysis of LCOGS domains

**Figure 3:**
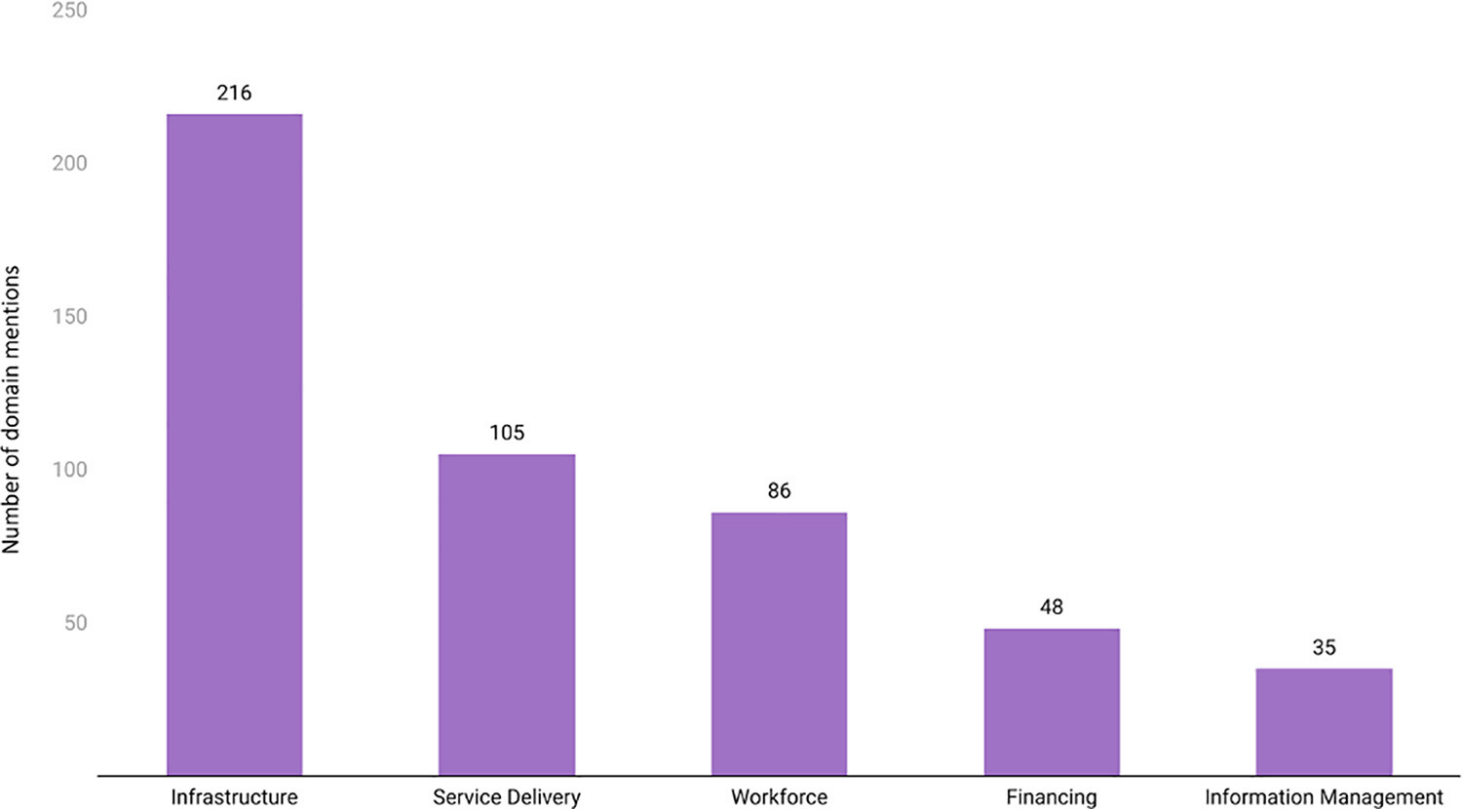
Distribution of SOTA care‑related mentions across five LCoGS domains.

The five LCoGS domains had 490 SOTA care‑related mentions. The most number of keyword mentions were under the infrastructure domain (216), while information management had the least number of mentions (35) (Figure 3). Relatively, 44.08% of mentions were for infrastructure, followed by 21.43% for service delivery, 17.55% for workforce, 9.79% for financing, and 7.14% for information management.

**Figure 4:**
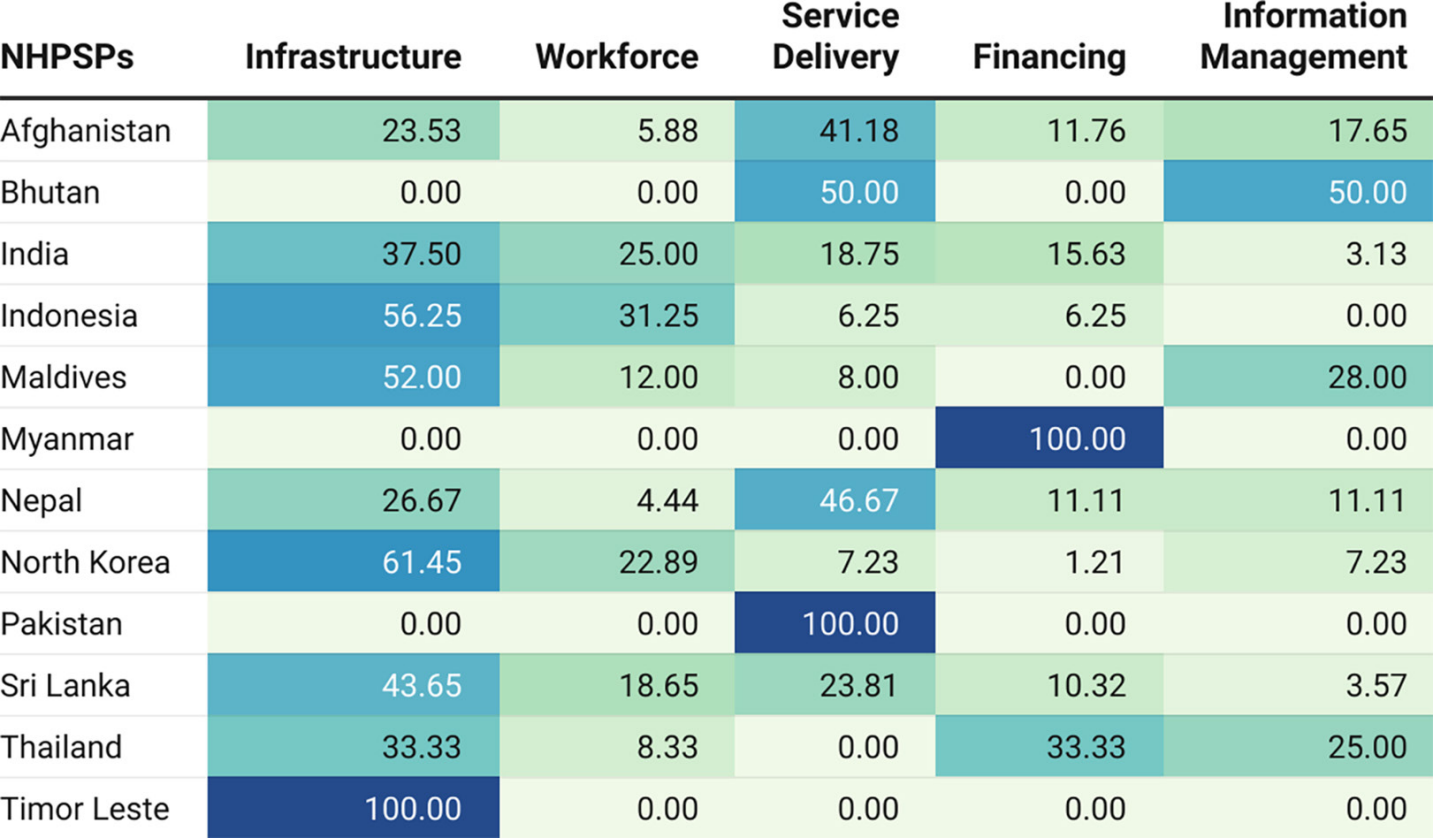
SOTA care‑related mentions across LCoGS domains in NHPSPs of South and Southeast Asian countries. Cell counts note the percentage of mentions of SOTA care‑related keywords.

Among countries, Sri Lanka had the highest number of mentions (252) across domains while Timor Leste had one mention for infrastructure, Myanmar had a mention under financing, and Pakistan had a mention under service delivery. Figure 4 notes the relative prioritization of domains for all countries. Afghanistan and Nepal included all domains with the greatest prioritization of service delivery and the least prioritization of workforce. Bhutan had no mentions under infrastructure, workforce, or financing domains. India and Sri Lanka included all domains with the greatest focus on infrastructure and the least on information management. Similar to India, Indonesia had a greater skew toward relative prioritization of infrastructure. The Maldives prioritized infrastructure the most with no mentions under financing. North Korea prioritized infrastructure the most and financing the least. Thailand equally prioritized infrastructure and financing with no mentions under service delivery.

## Discussion

### Summary of findings

This systematic and comprehensive analysis of the latest NHPSPs of 12 South and Southeast Asian countries found that relative prioritization of SOTA care, captured using mentions per keyword, was about four times lower compared with non‑SOTA care health issues. Eight of the twelve countries had lower SOTA care prioritization than non‑SOTA care‑related issues. Within SOTA care, pediatric surgery, anesthesia, and eye care receive negligible attention. Across LCoGS domains, infrastructure and service delivery were prioritized, while financing and information management remained unattended.

### Contextualization and policy implications

Prior research on SOTA care prioritization offers valuable context for our findings. Analysis of 43 NHPSPs in sub‑Saharan African countries found that 19% (8/43) of documents lacked any mention of SOTA care [35]. A previous analysis of forty different national‑level policy documents beyond the NHPSPs over seven decades in India noted that 5% of the documents had no SOTA care mentions [6]. Contrary to these, all NHPSPs of the twelve South and Southeast Asian countries included here had at least one SOTA care‑related mention. Focus across keywords, SOTA care subgroups, and LCoGS domains is also different between sub‑Saharan Africa versus South and Southeast Asia. For instance, “cancer” was mentioned more frequently in sub‑Saharan African NHPSPs than “surg*,” while this was not the case with South and Southeast Asia. Such differences are driven by differences in population health needs, policy processes, and other factors going beyond public health.

Differences notwithstanding, aspects of neglect toward SOTA care issues remain consistent. Evaluation of NHPSPs of 124 LMICs noted that only 7.3% of documents had any mentions related to child surgery [5]. India’s comprehensive policy analysis found that only two out of the forty national‑level documents in the last 70 years mention pediatric surgery [6]. More starkly, we found no pediatric surgery mentions in the twelve South and Southeast Asian NHPSPs. Such a lack of prioritization is alarming given that these regions contribute to >30% of the global population of children and adolescents [36]. Several of these countries have made steady progress on child mortality indicators in the last two decades relying on immunization and other public health interventions. Any further progress would require investing in conditions amenable to SOTA care interventions for children [37]. Policy focus is critical as it can determine downstream initiatives. For instance, the lack of focus on pediatric surgery in NHPSPs in LMICs is reflected in the limited inclusion of child surgery in NSOAPs of the sub‑Saharan African countries [38].

For LCoGS domains, the lower prioritization of information management and financing than infrastructure, workforce, or service delivery is also common between the current analysis and those involving NHPSPs from sub‑Saharan Africa. This lacuna in policy prioritization has direct implications. For instance, countries with NSOAPs struggle with implementation. In part, their challenges could be attributed to limited focus on SOTA care financing. Except for some data from India and Pakistan [39, 40], South and Southeast Asian countries have not systematically assessed their SOTA care indicators at the subnational (state, province, etc.) levels. Among other roadblocks [41], lack of prioritization of information management NHPSPs can potentially explain limited SOTA care indicators data collection. In 2015, all South and Southeast Asian countries (including Bangladesh, which we could not include in the analysis) signed the World Health Assembly Resolution WHA68.15 on Strengthening Emergency and Essential Surgical Care and Anesthesia as a Component of Universal Health Coverage [42]. Yet, SOTA care inclusion in NHPSPs is limited.

SOTA care is intimately connected with health systems strengthening. Solving the access disparities would lead to positive spillover effects in society [43, 44]. Integrating it into the NHPSPs would be the appropriate policy and planning choice. However, the lack of SOTA care prioritization in NHPSPs may point to the need for dedicated policy and planning initiatives like the NSOAP in South and Southeast Asian countries. As of 2024, Pakistan is the only South Asian country with an NSOAP‑like document known as the National Vision for Surgical Care [23, 45]. Pakistan had small differences in the MPK values for SOTA and non‑SOTA groups. This might partly explain Pakistan’s policy readiness for an NSOAP. Nepal is in the process of drafting an NSOAP [46]. Other countries currently lack public information on NSOAP development and implementation.

### Strengths, limitations, and future directions

This study incorporates the most recent and currently active National Health Policies, Strategies, and Plans from 12 countries in the SAARC and WHO‑SEARO groups. The chosen sets of keywords encompass various aspects of SOTA care, drawing upon established research in the field. Further, the use of non‑SOTA keywords related to important public health issues such as tuberculosis, human immunodeficiency virus/acquired immunodeficiency syndrome (HIV/AIDS), immunization, etc. present controls. The use of mentions per keyword allows for cross‑country comparisons and benchmarks our findings against previous research. Analysis of LCoGS domains and SOTA care subgroups helps identify critical areas demanding immediate attention.

We acknowledge several limitations, some of which can be resolved in the future. First, we could not incorporate Bangladesh’s NHPSP since we could not find an English version of the document on the WHO Country Planning Cycle Database and several relevant government websites. Second, our domain analysis assigned the mentions to domains where there was an explicit reference to SOTA care. This conservative approach may have reduced the mentions that we considered. For instance, terms like “cancer” and “blood” came up for SOTA care and non‑SOTA care contexts and we chose only those mentions where the context (i.e., the accompanying text) explicitly mentioned SOTA care issues. Future research could attempt a more lenient analysis. Third, we did not include the subnational (state or province) policies, strategies, and plans. However, based on our experience with previous comprehensive analysis in India [6], typically sub‑national planning tends to follow national policymaking and planning for issues like SOTA care that require dedicated financial investments across secondary and tertiary care hospitals. States or provinces often have to rely on national governments for such healthcare investments. So, they tend to align themselves with national policymaking and planning in a top‑down fashion. If national policies, strategies, and plans do not focus on SOTA care, then it is unlikely that state‑level policies, strategies, and plans would focus on SOTA care issues. While the inability to include the sub‑national documents is a limitation of our study, it is not a major limitation since the sub‑national policy documents, even if we had analyzed them, probably would not have included much information on SOTA care, knowing how little SOTA care was prioritized in the NHPSPs of several countries. Given that accessing subnational policy and planning documents is challenging, previous cross‑country evaluations of SOTA care prioritization have often limited themselves to national‑level documents [5, 35]. Lastly, relying solely on keyword mentions limits the assessment of policy prioritization as it leaves out broader healthcare issues that may be related to SOTA care distally. Future studies could improve our approach and validate it against objective metrics like disease burden or LCoGS indicators.

## Conclusion

Prioritizing SOTA care is integral to achieving UHC, especially in South and Southeast Asia where the current National Health Policies, Strategies, and Plans have a limited emphasis on their financing and information management. In addition to integrating SOTA care in NHPSPs, dedicated initiatives such as NSOAPs might be helpful in some countries in improving access. Such policy and planning focus aligns with the goals of global surgery and health systems strengthening, promoting better SOTA care for all, and fostering a more equitable healthcare landscape in South and Southeast Asia.

## Data Availability

All data used and produced in the manuscript can be requested from the authors.
